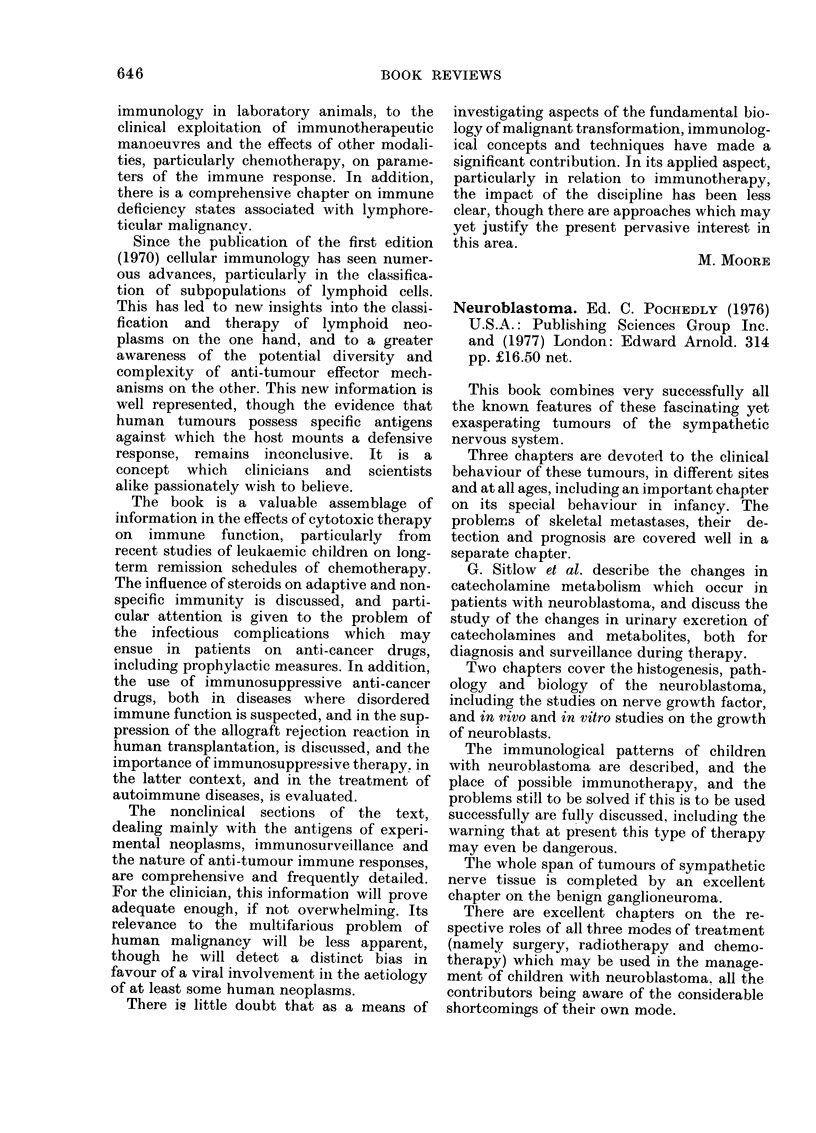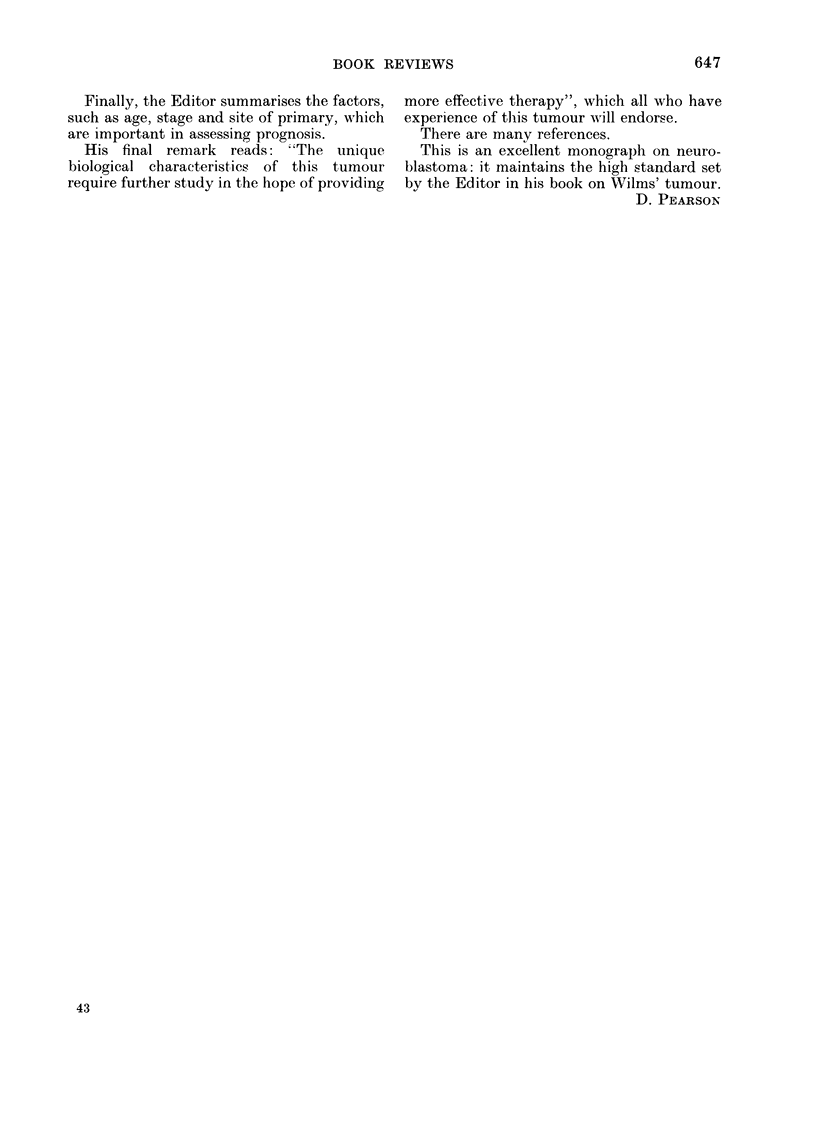# Neuroblastoma

**Published:** 1977-11

**Authors:** D. Pearson


**Neuroblastoma.** Ed. C. POCHEDLY (1976) U.S.A.: Publishing Sciences Group Inc. and (1977) London: Edward Arnold. 314 pp. £16.50 net.

This book combines very successfully all the known features of these fascinating yet exasperating tumours of the sympathetic nervous system.

Three chapters are devoted to the clinical behaviour of these tumours, in different sites and at all ages, including an important chapter on its special behaviour in infancy. The problems of skeletal metastases, their detection and prognosis are covered well in a separate chapter.

G. Sitlow *et al.* describe the changes in catecholamine metabolism which occur in patients with neuroblastoma, and discuss the study of the changes in urinary excretion of catecholamines and metabolites, both for diagnosis and surveillance during therapy.

Two chapters cover the histogenesis, pathology and biology of the neuroblastoma, including the studies on nerve growth factor, and *in vivo* and *in vitro* studies on the growth of neuroblasts.

The immunological patterns of children with neuroblastoma are described, and the place of possible immunotherapy, and the problems still to be solved if this is to be used successfully are fully discussed, including the warning that at present this type of therapy may even be dangerous.

The whole span of tumours of sympathetic nerve tissue is completed by an excellent chapter on the benign ganglioneuroma.

There are excellent chapters on the respective roles of all three modes of treatment (namely surgery, radiotherapy and chemotherapy) which may be used in the management of children with neuroblastoma, all the contributors being aware of the considerable shortcomings of their own mode.

Finally, the Editor summarises the factors, such as age, stage and site of primary, which are important in assessing prognosis.

His final remark reads: "The unique biological characteristics of this tumour require further study in the hope of providing more effective therapy", which all who have experience of this tumour will endorse.

There are many references.

This is an excellent monograph on neuroblastoma: it maintains the high standard set by the Editor in his book on Wilms' tumour.

D. Pearson